# Population Diversity among *Bordetella pertussis* Isolates, United States, 1935–2009

**DOI:** 10.3201/eid1808.120082

**Published:** 2012-08

**Authors:** Amber J. Schmidtke, Kathryn O. Boney, Stacey W. Martin, Tami H. Skoff, M. Lucia Tondella, Kathleen M. Tatti

**Affiliations:** Centers for Disease Control and Prevention, Atlanta, Georgia, USA

**Keywords:** whooping cough, Bordetella pertussis, pertussis, genetic diversity, molecular typing, wP, aP, acellular vaccines, bacteria, United States, vaccination, immunization, alleles, isolates

## Abstract

Since the 1980s, pertussis notifications in the United States have been increasing. To determine the types of *Bordetella pertussis* responsible for these increases, we divided 661 *B. pertussis* isolates collected in the United States during 1935–2009 into 8 periods related to the introduction of novel vaccines or changes in vaccination schedule. *B. pertussis* diversity was highest from 1970–1990 (94%) but declined to ≈70% after 1991 and has remained constant. During 2006–2009, 81.6% of the strains encoded multilocus sequence type *prn2-ptxP3-ptxS1A-fim3B*, and 64% were multilocus variable number tandem repeat analysis type 27. US trends were consistent with those seen internationally; emergence and predominance of the *fim3B* allele was the only molecular characteristic associated with the increase in pertussis notifications. Changes in the vaccine composition and schedule were not the direct selection pressures that resulted in the allele changes present in the current *B. pertussis* population.

## Abstract 2

Resurgence of pertussis was not directly correlated with changes in vaccine composition or schedule.

Pertussis, or whooping cough, is caused by the bacterium *Bordetella pertussis* and is the most frequently reported bacterial vaccine-preventable disease in the United States (*1*). Vaccination against pertussis began in the 1940s in the United States, using a whole-cell formulation (wP) that resulted in a dramatic decrease in infections and deaths (*2*). Acellular pertussis vaccines (aP) were licensed for the fourth and fifth doses of the childhood booster series in 1991 and were recommended for all 5 doses of the childhood series by 1997; in 2005, a single-dose adolescent and adult booster (tetanus-diphtheria-aP, or Tdap) was recommended (Figure 1). Despite a successful US childhood vaccination program with high coverage, the number of reported pertussis cases has increased since the early 1980s, with 27,550 cases reported in 2010 (*3*).

**Figure 1:**
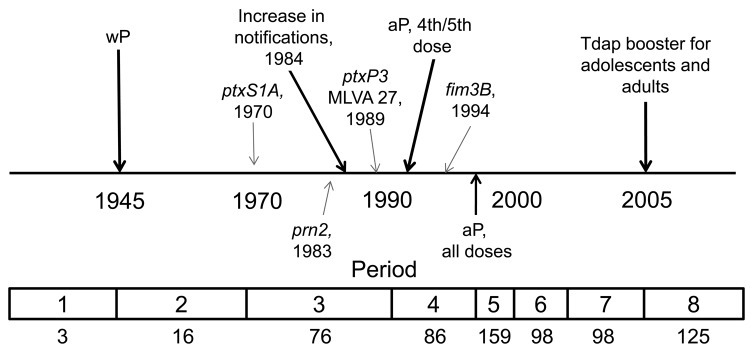
Timeline of pertussis vaccine introduction in the United States and appearance of alleles within the *Bordetella pertussis* population, 1935–2009. The 8 periods used in this study are indicated at bottom; numbers below indicate number of selected strains during that period (N = 661). wP, whole-cell pertussis vaccine; MLVA 27, multilocus variable number tandem repeat analysis type 27; aP, acellular pertussis vaccine; Tdap, tetanus-diphtheria-aP.

Before the current study, US *B. pertussis* isolates from 1935–1999 were characterized by pulsed-field gel electrophoresis (*4*), and a subset of isolates was analyzed for 2 genes, *prn* and *ptxS1* (*5*). Genetically, the *B. pertussis* population was largely homogeneous during this period, and only a few strain types caused most disease in the United States (*4*). Recently, the molecular typing methods multilocus variable number tandem repeat analysis (MLVA) and multilocus sequence typing (MLST) have been used to assess *B. pertussis* population trends in other countries (*6**–**9*); used together, these methods offer discriminatory power similar to that of pulsed-field gel electrophoresis (*9**,**10*). We used MLVA and MLST to type a large selection of *B. pertussis* isolates from the United States and examined a selection of molecular changes that occurred over time and how these changes related to increases in pertussis notifications or changes in vaccine policy.

## Methods

### Strain Selection

We selected 661 *B. pertussis* isolates of US origin from the Centers for Disease Control and Prevention (CDC) collection by using random sampling stratified by geography (US states and territories) and period. The strains were divided in advance as follows: period 1 (prevaccine era), 1935–1945, n = 3; period 2 (early wP era), 1946–1969, n = 16; period 3 (late wP era), 1970–1990, n = 76; period 4 (aP transition for 4th and 5th dose of childhood series), 1991–1996, n = 86; period 5 (early aP), 1997–1999, n = 159; period 6 (middle aP), 2000–2002, n = 98; period 7 (late aP), 2003–2005, n = 98; and period 8 (early Tdap booster), 2006–2009, n = 125 (Figure 1). Stratification was used to ensure that all states and territories with isolates in the strain bank were represented in the random sample. The geographic distribution of strains and information regarding location and year of isolation are provided in the Technical Appendix. We could not correct for the lack of representativeness of the isolates in the collection because CDC does not receive an isolate for every report of illness in the United States. After 3 days of incubation at 37°C, DNA was extracted by heat-lysis preparation from each isolate and stored at −20°C until ready for use in PCR.

### MLVA

Analysis was performed by using a 6-target multiplex similar to that described (*8*) with some modifications. Using the HotStarTaq kit (QIAGEN, Valencia, CA, USA) yielded a final reaction volume of 20 µL. Master mix 1 consisted of fluorescently labeled oligonucleotides for variable number tandem repeats (VNTRs) 1 (0.13 µmol/L each primer), 5 (0.09 µmol/L each primer), and 6 (0.09 µmol/L each primer) and was supplemented with 1 mol/L betaine (Sigma-Aldrich, St. Louis, MO, USA) to facilitate primer–template interaction. Master mix 2 consisted of fluorescently labeled oligonucleotides for VNTRs 2 (0.08 µmol/L each primer), 3 (0.23 µmol/L each primer), and 4 (0.08 µmol/L each primer) and was supplemented with 10% dimethyl sulfoxide (Sigma-Aldrich). PCR was performed in single-target reactions (*6*) for some targets that were not efficiently amplified by using the multiplex assay format. Amplified products were diluted 1:50 and 1:100 and mixed with 0.5 µL MapMarker X-Rhodamine labeled 400-bp ladder (BioVentures, Murfreesboro, TN, USA). Sizes were determined by using the ABI Prism 3130xl Genetic Analyzer (Applied Biosystems, Foster City, CA, USA); VNTR sizes were determined by using GeneMapper version 4.0 software (Applied Biosystems). Sizing data for all strains were compared with those found for the *B. pertussis* prototype strain, Tohama I, to determine the repeat count for each locus. The assignment of an MLVA type was based on the combination of repeat counts for VNTRs 1, 3a, 3b, 4, 5, and 6 and was consistent with international nomenclature. Novel MLVA combinations were submitted to the laboratory of Frits Mooi (National Institute for Public Health and the Environment, Bilthoven, the Netherlands) for MLVA type designation.

### MLST

Our algorithm consisted of 4 DNA targets: the pertactin (*prn*) gene, the first gene in the pertussis toxin operon and its respective promoter (*ptxP*-*ptxS1*), and the fimbrial protein-encoding gene (*fim3*). The *prn* and *fim3* genes were amplified by using oligonucleotides and conditions as described (*6**,**11*). The *ptxP-ptxS1* region was amplified by using oligonucleotides Ptox1Fpert (5′-CCCTCGATTCTTCCGTACATCC-3′) and Ptox2R (5′-CGCGATGCTTTCGTAGTACA-3′), resulting in an amplified product of 964 nt. Products were sequenced and analyzed as described (*10*).

### Population Analysis

Typing data among strains were compiled by using BioNumerics software version 5.01 (Applied Maths, Sint-Martins-Latem, Belgium). Minimum spanning trees (MSTs) were generated by using default settings and the Manhattan coefficient. The Simpson index of diversity (DI) and 95% CIs were calculated as described by Hunter and Gaston (*12*) and Grundmann et al. (*13*), respectively. DI was calculated by using a combination of MLVA + MLST to define types. For example, MLVA 27-*prn2-ptxP3-ptxS1A-fim3A* was considered a unique type from MLVA 27-*prn2-ptxP3-ptxS1A-fim3B*. DI is represented as 1 – *D* × 100 so that the level of diversity is proportional to the percentage. The Pearson correlation coefficient (r) was used to detect linear dependence between pertussis notifications and predominant molecular changes.

## Results

### Identification of Strains using MLVA + MLST

**Figure 2:**
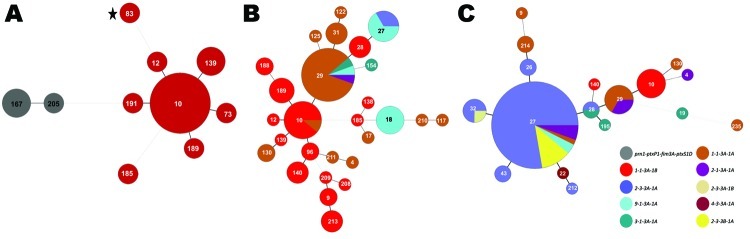
Minimum spanning trees depicting changes within the *Bordetella pertussis* population, United States, 1935–1996. Multilocus variable number tandem repeat analysis (MLVA) types are represented by circles and are scaled to member count within each panel; multilocus sequence typing (MLST) types are represented by color. A) Periods 1 and 2, 1935–1945 (n = 3) and 1946–1969 (n = 16), respectively. These 2 periods were combined for the generation of the tree. Period 1 (prevaccine era) strains are shown on the left, distantly related to period 2 strains (right side) from the early whole-cell pertussis vaccine (wP) era. B) Period 3, 1970–1990, n = 76. During this period, when wP was in use, a high degree of diversity was identified; 2 predominant MLST types differed by the *ptxS1* allele. C) Period 4, 1991–1996, n = 86. During the transition from wP to acellular pertussis vaccine for the 4th and 5th dose of the childhood series, MLVA 27, *ptxP3*, and *prn2* were dominant, and the *fim3B* allele emerged.

The prevaccine era (period 1, 1935–1945) is depicted in Figure 2, panel A, left side; all 3 strains encoded the same MLST profile, *prn1-ptxP1-ptxS1D-fim3A*. The strain identified in Figure 2, panel A, as MLVA 167 is the 10536 strain used in the manufacture of the Sanofi-Pasteur (Swiftwater, PA, USA) aP in the United States (*7*). Neither the MLVA types found (167 or 205) nor the MLST profile for period 1 are seen again in later periods. The dotted line between MLVA circles 191 and 167 in Figure 2, panel A, indicates a distant genetic relationship between the respective clusters for periods 1 and 2. The predominant MLVA type during period 2 (1946–1969), the early wP era, was 10 (Figure 2, panel A, right side), and the MLST profile shifted to *prn1-ptxP1-ptxS1B-fim3A*, identical to Tohama I (identified as MLVA circle 83 with a star), representing a single-locus change to *ptxS1B* compared with period 1. The strain used for manufacture of the GlaxoSmithKline (Research Triangle Park, NC, USA) pertussis vaccine in the United States is Tohama I.

Many MLVA and MLST types were found among the 76 strains in period 3 (1970–1990), the late wP era (Figure 2, panel B). MLVA 10 was still present from period 2, but other types also dominated, including MLVA 29. MLVA 27, the dominant type among isolates from period 8, emerged in 2 strains from Ohio and 2 from Missouri isolated in 1989. Many of the strains characterized in period 3 differed from period 2 in *ptxS1* by encoding the A allele, which was first observed in a 1970 isolate from Colorado. In addition, the first *prn2* allele was found in a 1983 isolate from Washington, DC, whereas the *ptxP3* allele was first characterized in an Ohio isolate from 1989 (Figure 1). The 1989 isolate from Ohio was the first in our random selection of US isolates that encoded the combined typing data of MLVA 27 with *prn2-ptxP3-ptxS1A-fim3A* (Figure 2, panel B). This MLST pattern was dominant for the subsequent 2 periods and represents a single-locus intermediary to the *prn2-ptxP3-ptxS1A-fim3B* MLST pattern (dominant in period 8).

The MST of 86 strains from period 4 (1991–1996) is shown in Figure 2, panel C. The aP vaccine was recommended for the fourth and fifth doses of the childhood series in 1991. During this time, MLVA 27, *ptxP3*, and *prn2* were dominant. The *fim3B* allele was first noted in an Idaho isolate from 1994. The *prn1-ptxP1-ptxS1B-fim3A* MLST type that was widely distributed throughout periods 2 and 3 was restricted to 8% of the selected strains during period 4 and corresponded with MLVA 10 (also observed in periods 2 and 3).

**Figure 3:**
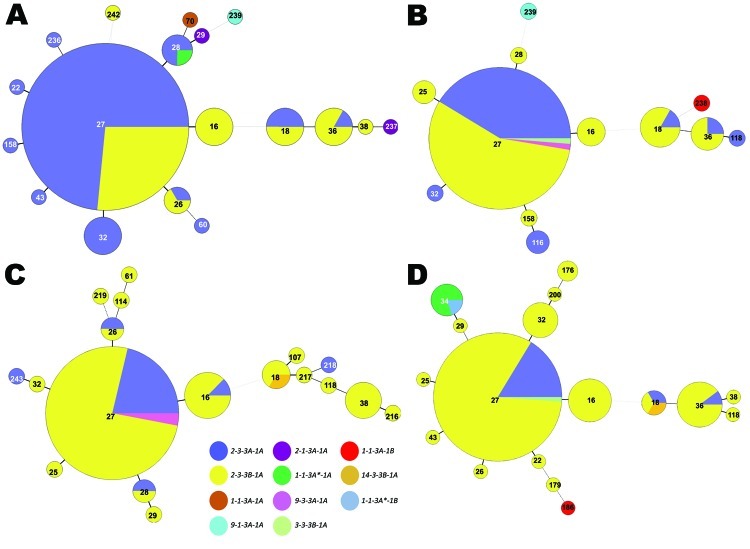
Minimum spanning trees depicting changes within the *Bordetella pertussis* population, United States, 1997–2009. Multilocus variable number tandem repeat analysis (MLVA) types are represented by circles and are scaled to member count within each panel; multilocus sequence typing (MLST) types are represented by color. A) Period 5, 1997–1999, n = 159, the early years of acellular pertussis vaccine (aP) use. B) Period 6, 2000–2002, n = 98. With aP in use, MLVA 27 with the *fim3B* allele dominated. C) Period 7, 2003–2005, n = 98. In 2004, during the late aP use period, the novel pertactin allele (*prn14*) was identified in an isolate from New York. D) Period 8, 2006–2009, n = 125. After the introduction of the aP booster for adolescents and adults, MLST type *prn1-ptxP1-ptxS1B-fim3A* (previously found in periods 2–4 and 6) reappeared with a new MLVA type.

**Figure 4:**
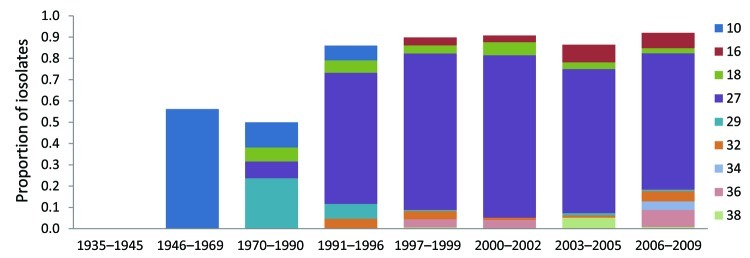
Frequency (by proportion of all isolates tested) of predominant multilocus variable number tandem repeat analysis (MLVA) types within the *Bordetella pertussis* population, United States, 1935–2009. MLVA 10 was dominant in period 2 (1946–1969) but decreased through periods 3 (1970–1990) and 4 (1991–1996) while MLVA 18, 27, and 29 emerged. MLVA 27 increased in proportion during period 4 and dominated the population for the rest of the study period; however, the proportion of MLVA 27 has been decreasing since period 6 (2000–2002), allowing for the emergence of other types.

Period 5 (1997–1999) is depicted in Figure 3, panel A. The aP was recommended for all 5 doses of the childhood immunization series in 1997. MLVA 27 increased to 73.6% of the strains compared with 62.1% for period 4 (Figure 4). Diversity constricted to a total of 7 MLST types (10 were seen previously). The fim3A* allele was identified in 1999 in a New Hampshire isolate and is shown in green within the MLVA 28 circle in Figure 3, panel A. The proportion of the population that encoded *prn2-ptxP3-ptxS1A-fim3B* (Figure 2, Figure 3) increased from 6.9% in period 4 to 30.8% in period 5.

Figure 3, panel B, shows the MST for the mid-aP era (period 6). MLVA 27 and the *prn2-ptxP3-ptxS1A-fim3B* profile continued to dominate. However, MLVA 27 represented 76.5% of selected strains, thus reaching a plateau in frequency. Meanwhile, the *prn2-ptxP3-ptxS1A-fim3B* MLST profile increased to represent 58% of strains. The MLST type *prn1-ptxP3-ptxS1B-fim3A* that was previously found in periods 2–4 reappeared with a new MLVA type, 238.

The late-aP era (period 7) is shown in Figure 3, panel C. During this time, a novel pertactin allele (*prn14*; GenBank accession no. HQ165753) was identified in an isolate from New York in 2004, shown in light orange in Figure 3, panel C. MLVA 27 decreased to 67.3% of the strains while the yellow MLST profile (*prn2-ptxP3-ptxS1A-fim3B*) increased to 77.6%.

The MST for period 8 is shown in Figure 3, panel D. The Tdap booster for adolescents and adults was recommended for use in 2005. MLVA 27 frequency remained approximately the same as for period 7, 64%. The MLST profile *prn2-ptxP3-ptxS1A-fim3B* increased to 81.6% of the strains. Meanwhile, the *prn1* (n = 6), *ptxP1* (n = 6), and *ptxS1B* (n = 2) alleles reemerged; these alleles are also encoded by vaccine strain Tohama I. The last time these alleles were seen in multiple strains was in period 4, with 17 strains encoding *prn1*, 28 strains encoding *ptxP1*, and 8 strains encoding *ptxS1B*.

### Trends in Typing Data during 74 Years of US History

DI values and 95% CIs are provided in the Table. During periods 1 and 2, the DI was in the mid to upper 60% range, but the CIs were large due to a low sample size. Period 3 (1970–1990) had a DI of 94.0% with a small CI that was distinct from previous time periods. To determine if the length of the time interval (20 years) was biasing the results, period 3 was subdivided into 5- and 10-year intervals; all DI values remained ≈90% with small 95% CIs. DI decreased to 75.7% in period 4 and remained relatively constant following the introduction of aP.

**Table:** Diversity trends among selected *Bordetella pertussis* isolates, United States, 1935–2009*

Study period	Years	Vaccine use	Simpson diversity index, % (95% CI)
1	1935–1945	Pre-wP	66.7 (30.4–103.0)
2	1946–1969	Early-wP	77.8 (59.4–96.2)
3	1970–1990	Late-wP	94.0 (90.9–97.1)
4	1991–1996	wP/aP transition (4th–5th doses)	75.7 (65.9–85.5)
5	1997–1999	Early-aP	66.8 (59.5–74.1)
6	2000–2002	Mid-aP	70.2 (67.7–81.1)
7	2003–2005	Late-aP	71.6 (62.4–80.8)
8	2006–2009	Tdap	69.3 (60.7–77.9)

*wP, whole cell vaccine; aP, acellular vaccine; Tdap, tetanus-diphtheria-aP.

**Figure 5:**
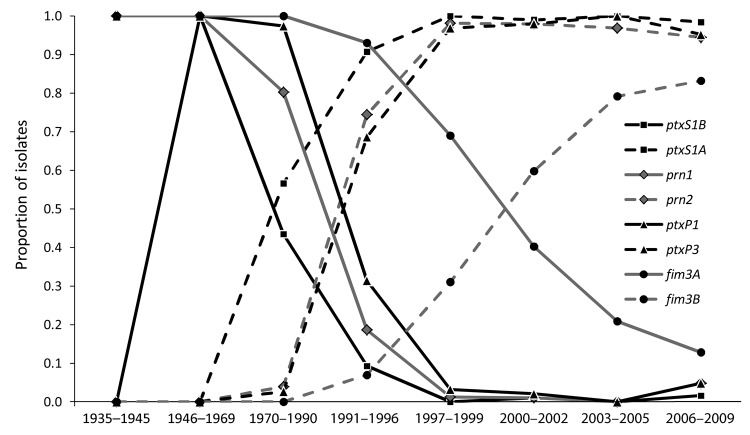
Transitions of frequency (by proportion of all isolates tested) of dominant alleles for each multilocus sequence typing (MLST) type target within the *Bordetella pertussis* population, United States, 1935–2009. The previous dominant type is denoted by a solid line, with the new dominant type denoted by a dashed line of the same style. The dashed lines of *prn2* and *ptxP3* overlap with each other and multilocus variable number tandem repeat analysis (MLVA) type 27 (Figure 6), which suggests they arose at approximately the same time and resulted in the new dominant MLVA + MLST profile. The transition from *fim3A* to *fim3B* occurred much later than the other transitions.

The frequencies of individual MLST alleles and MLVA types over time are shown in Figure 5. Changes in *ptxS1* occurred first with the transition of *ptxS1B* to *ptxS1A* beginning in the 1970s (first observed in a 1970 isolate from Colorado). Later, changes within *prn*, *ptxP*, and MLVA 27 occurred at approximately the same time, with transitions to MLVA 27, *prn2*, and finally *ptxP3*. In 1995, *ptxS1A* was encoded by 100% of tested strains, and in 1996, >90% of strains tested encoded *prn2* or *ptxP3*, but more recently, frequency of *prn2* and *ptxP3* has declined (period 8). The transition within *fim3* observed in the early 2000s was a more gradual increase that did not approach 100% as with the other MLST alleles.

### Comparison of Typing Data and Increase in US Pertussis Notifications

**Figure 6:**
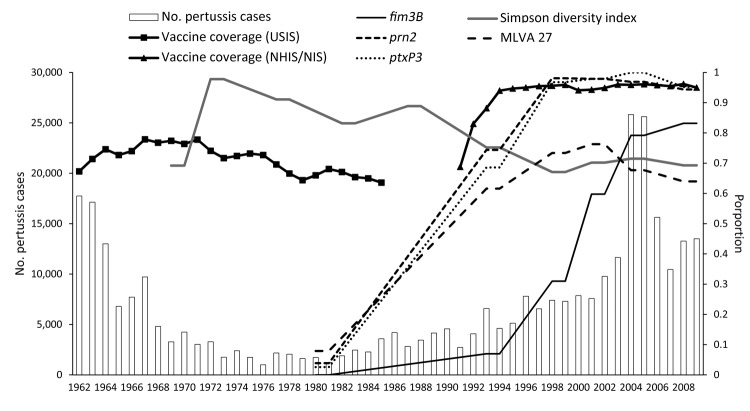
Comparison of number of pertussis notifications, proportion of vaccine coverage, and proportion of dominant multilocus sequence typing alleles and multilocus variable number tandem repeat analysis (MLVA) type 27 among a random selection of 661 isolates, United States, 1935–2009. Bars indicate case notifications; lines indicate 2-point moving average distributions of frequency for the time periods assigned in this study. Vaccine coverage data were collected for the United States Immunization Survey (USIS, 1962–1985), National Health Interview Survey (NHIS, 1991−1993), and National Immunization Survey (NIS, 1994−2009). No data are available for 1986−1990 because USIS was cancelled (*15*). The *fim3B* trend line was temporally and significantly associated with the rate of increase for pertussis notifications.

We aligned annual US pertussis notifications with vaccine coverage data, the trend lines for each of the dominant MLST alleles, MLVA 27, and DIs (Figure 6). An inverse relationship was observed between DI and notifications as well as vaccine coverage. Increases among *ptxS1A*, *prn2*, *ptxP3*, and MLVA 27 were not significantly correlated with the increase in notifications, whereas the proportion of *fim3B*-encoding strains was significantly correlated with pertussis notifications (r = 0.8608; p = 0.0277).

## Discussion

Changes in the US *B. pertussis* population have followed trends that are largely consistent with other nations and yet were unique in the correlation of annual case counts with *fim3B*. Our findings regarding DI trends were similar to those found for the United Kingdom (*9*), but unlike a previous study that examined *B. pertussis* in the Netherlands (*14*), we did not find a correlation between the *ptxP3* allele and annual reporting of pertussis cases in the United States.

MLVA + MLST analysis showed that the US *B. pertussis* population changed genetically during the period covered by our study (Figure 2, Figure 3). We found a high degree of diversity in period 3 (1970s and 1980s), with a DI of 94%, that was consistent with findings (84%) for a similar period in the United Kingdom (*7*). The results from the United Kingdom were attributed to a decline in vaccine coverage rates below 80% during 1975–1989, with a low of 31% in 1978 (*7*). Parent-supplied data from the US Immunization Survey for 1962–1985 showed US coverage among 2-year-old children for >3 doses of wP declined from a peak of 77.9% in 1967 to a low of 63.6% in 1985; vaccination rates then increased dramatically, to >94% by 1994, and have remained high ever since (Figure 6) (*15*).

Given that *B. pertussis* has no nonhuman hosts or environmental niche, vaccine-mediated immunity is the most likely selective pressure against *B. pertussis*. Therefore, vaccination coverage may have contributed to the increase in diversity during period 3. This hypothesis also supports the correlation between the decline in diversity as the rate of vaccination coverage (>3 doses) increased to ≈95% in the mid-1990s. The emergence and subsequent dominance of MLVA 27 and the MLST alleles *prn2* and *ptxP3* (in that order, temporally) in the United States occurred during the period with the highest diversity, period 3.The timing and order of these transitions are consistent with global trends (*16*) in which the emergence of nonvaccine-type alleles for *ptxS1* and *prn* appeared 15–30 years after the introduction of pertussis vaccines. Despite increasing pertussis incidence in the United States, diversity has remained stable for *B. pertussis* during the past 20 years; a similar trend was observed in the Netherlands (*6*).

Regional clustering of *B. pertussis* MLVA types may be best exemplified by comparing the United States to Australia. Kurniawan et al. found that a single MLVA type, 70, emerged in Australia’s wP era and dominated during the aP transition (reaching ≈40% of isolates), then declined once the transition to aP was complete (*8*). A similar rise and fall of MLVA 70 surrounded the transition to aP in the United Kingdom, where MLVA 70 disappeared after 2004 (*7*). However, to our knowledge, MLVA 70 has only been detected once in the United States, in a Missouri isolate from 1997. Further, MLVA 64, which represents ≈10% of the *B. pertussis* population in Australia, was not detected in the United States. Our findings, combined with the findings from the United Kingdom, do not support the conclusion that the introduction of aP was the driving factor responsible for the emergence and dominance of MLVA 70 or 64. On the contrary, the data suggest that aP helped to eliminate MLVA 70. Whole-genome sequencing has implicated regional bottlenecks as a likely contributor to the geographic restriction of particular MLVA types (*17**,**18*), which may explain the exclusive prevalence of MLVA 70 in Australia (*8*). Furthermore, the timing of the emergence and dominance of the MLVA 27 and MLST alleles *prn1*, *ptxS1A*, and *ptxP3* in the United States predate the completion of the wP to aP transition (1997) by ≈10 years (Figure 1, Figure 2, Figure 3, Figure 5, Figure 6). The emergence and dominance of the *fim3B* allele (Figure 6) probably coincides with the increase in notifications.

Many of the allele changes we found have been identified in clinical isolates of *B. pertussis* throughout the world. However, any conclusions involving individual gene loci in clinical isolates are vulnerable to phenotypic results that arise from mutations elsewhere in the genome. The clearest way to identify the effect of allele mutations is to examine them alone and in combination in a genetically controlled bacterial background (*19*). Therefore, it is premature to associate allele changes with a phenotype, disease severity, or events of epidemiologic importance until they are functionally analyzed individually and cumulatively in a model system. Data related to the functional or clinical effects of allele changes for the MLST targets used in this study are limited.

Recently, an increase in pertussis notifications and a 1.41-fold increase in hospitalizations were correlated with the increasing presence of the *ptxP3* allele in circulating *B. pertussis* isolates (*20*). Unfortunately, the *ptxP* allele was not characterized for *B. pertussis* among hospitalized patients, so a direct correlation could not be made between *ptxP3* and disease severity. In addition, ELISA was used to demonstrate a modest increase (1.6-fold) in pertussis toxin production among *ptxP3* strains relative to *ptxP1*-encoding strains when grown in vitro. In vivo experimentation using genetically controlled *B. pertussis* mutants for *ptxP3* is needed to determine whether a 1.6-fold increase is sufficient to cause more severe disease or to overwhelm vaccine-mediated anti–pertussis toxin antibody response. Bart et al. hypothesized that *ptxP3* may be a “hitchhiker” mutation that benefited from advantageous mutations selected elsewhere in the genome (*18*); our findings lend support to this hypothesis.

More information is known about the effects of the *prn* and *ptxS1* allele changes, but studies assessing the *fim3* locus are lacking. The divergence among the pertactin alleles is proximal to the encoded RGD motif that is involved in eukaryotic cell binding and antigen presentation to B cells (*21*). In theory, such mutations could biochemically affect protein folding, host cell binding, or recognition by B cells (*14*); this hypothesis is supported by the finding that the pertactin variants 1–3 induce type-specific antibodies (*22*). However, the effects of these insertions/deletions on the pertactin protein product have not been determined experimentally. The *ptxS1A* allele encodes 3 amino acid changes relative to the 10536 vaccine type, *ptxS1D*, and 1 amino acid change compared with *ptxS1B* for Tohama I–based vaccine (*23*). The pertussis toxin remains biologically functional despite these changes (*24*). In vivo, mouse-derived anti–pertussis toxin antibodies tolerate numerous amino acid substitutions in *ptxS1* with equal neutralization between wild-type and mutant isolates (*25*). Therefore, *ptxS1* allele changes may not be clinically or immunologically relevant.

Little is known about the functional or in vivo effects of the *fim3B* mutation on protein function, bacterial survival, and adherence. The *fim3B* allele results in an alanine-to-glutamic acid mutation at aa 87 (*11*). Biochemically, this is a potentially important residue change, and the mutation is located in a surface epitope of *fim3* (aa 79–91) that has been shown to interact with human serum (*26*). Given the significant correlation between the increases in *fim3B* and US pertussis case notifications, the effect of the *fim3* mutation needs to be functionally and clinically determined. Alternatively, this could be another example of a regional bottleneck (*17**,**18*); additional data regarding the prevalence of *fim3B* in other countries is needed to rule out this possibility. Moreover, the strain collection available for this study may not be fully representative of the *B. pertussis* population in the United States over time. Efforts were made to ensure that the strains selected for this study were diverse in year of isolation as well as geography within the United States. According to Mouillot (*27*), DI can be influenced by selection bias and sample size, but almost all studies evaluating a historical collection of strains encounter this limitation, including the recent study in the United Kingdom (*7*).

In summary, the US *B. pertussis* population has evolved in the time since vaccinations were introduced in the 1940s (Figure 2, Figure 3, Figure 4, Figure 5). Our findings demonstrate that the resurgence of pertussis in the United States was not correlated with the *ptxP3* allele but with the presence of the *fim3B* allele among the *B. pertussis* population. The commonly circulating strains of *B. pertussis* in the United States encode different alleles compared with the strains used for manufacture of the pertussis vaccines, but the relevance of these allele changes remains to be fully elucidated. Because *B. pertussis* has no nonhuman host, the selective pressures it encounters are limited to the human immune system and the vaccine, but the influence of this selection pressure versus natural evolution on the modern US *B. pertussis* population is unclear. For example, minor types are beginning to emerge, including the reemergence of vaccine-type alleles. In addition, the vaccine policies of other nations may have contributed to the makeup of the US *B. pertussis* population in ways that could not be measured by using this study of US-based isolates.

As vaccine coverage rates improve among adolescents and adults, changes in the *B. pertussis* population should be monitored through molecular typing. The in vivo effects of MLST allele changes in a genetically controlled model for pathogen and host should be characterized to determine what effects, if any, these allele changes have with respect to vaccine-mediated immunity to circulating *B. pertussis*. More specific studies, such as genomic sequencing of particular strains and genetic expression of the multiple alleles in animal models, should be performed to determine the virulence and pathogenesis of these variants.

Technical Appendix Geographic distribution of *Bordetella pertussis* strains, by location and year of isolation, United States, 1935–2009. Period 1 (prevaccine era), 1935–1945, n = 3; period 2 (early wP era), 1946–1969, n = 16; period 3 (late wP era), 1970–1990, n = 76; period 4 (aP transition for 4th and 5th dose of childhood series), 1991–1996, n = 86; period 5 (early aP), 1997–1999, n = 159; period 6 (middle aP), 2000–2002, n = 98; period 7 (late aP), 2003–2005, n = 98; and period 8 (early Tdap booster), 2006–2009, n = 125.
